# Supplementary material for: Transcriptome and metabolome profiling reveal the inhibitory effects of food preservatives on pathogenic fungi
Source: PeerJ. 2025 Jul 23;13:e19737. doi: 10.7717/peerj.19737 (PMC12296564; doi:10.7717/peerj.19737)
Supplement: Supplemental Information 1 [file peerj-13-19737-s001.pdf]

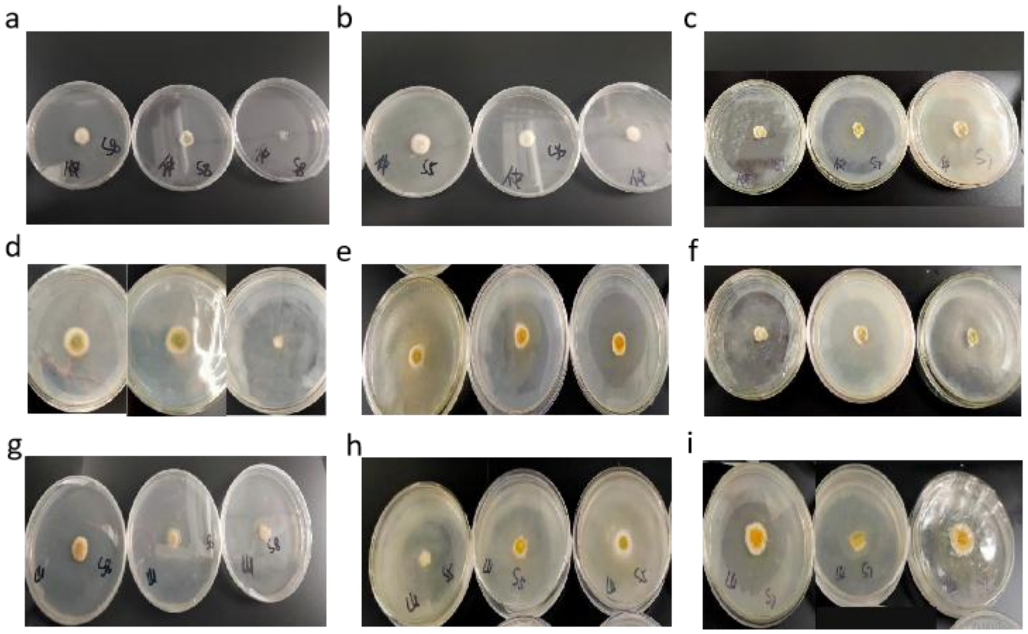

Figure S1. Growth inhibition of pathogenic bacteria by three preservation. (a) Growth inhibition of Aspergillus flavus by sec-butylamine; (b) Growth inhibition of Alternaria alternata by sec-butylamine; (c) Growth inhibition of Talaromyces funiculosus by sec-butylamine; (d) Growth inhibition of Aspergillus flavus by citric acid; (e) Growth inhibition of Alternaria alternata by citric acid; (f) Growth inhibition of Talaromyces funiculosus by citric acid; (g) Growth inhibition of Aspergillus flavus by potassium; (h) Growth inhibition of Alternaria alternata by potassium; (i) Growth inhibition of Talaromyces funiculosus by potassium sorbate.
