# Supplementary material for: Transcriptome and metabolome profiling reveal the inhibitory effects of food preservatives on pathogenic fungi
Source: PeerJ. 2025 Jul 23;13:e19737. doi: 10.7717/peerj.19737 (PMC12296564; doi:10.7717/peerj.19737)
Supplement: Supplemental Information 2 — Z1, Z2, and Z3 represented treatment samples, and ZC1, ZC2, and ZC3 represented control samples. [file peerj-13-19737-s002.pdf]

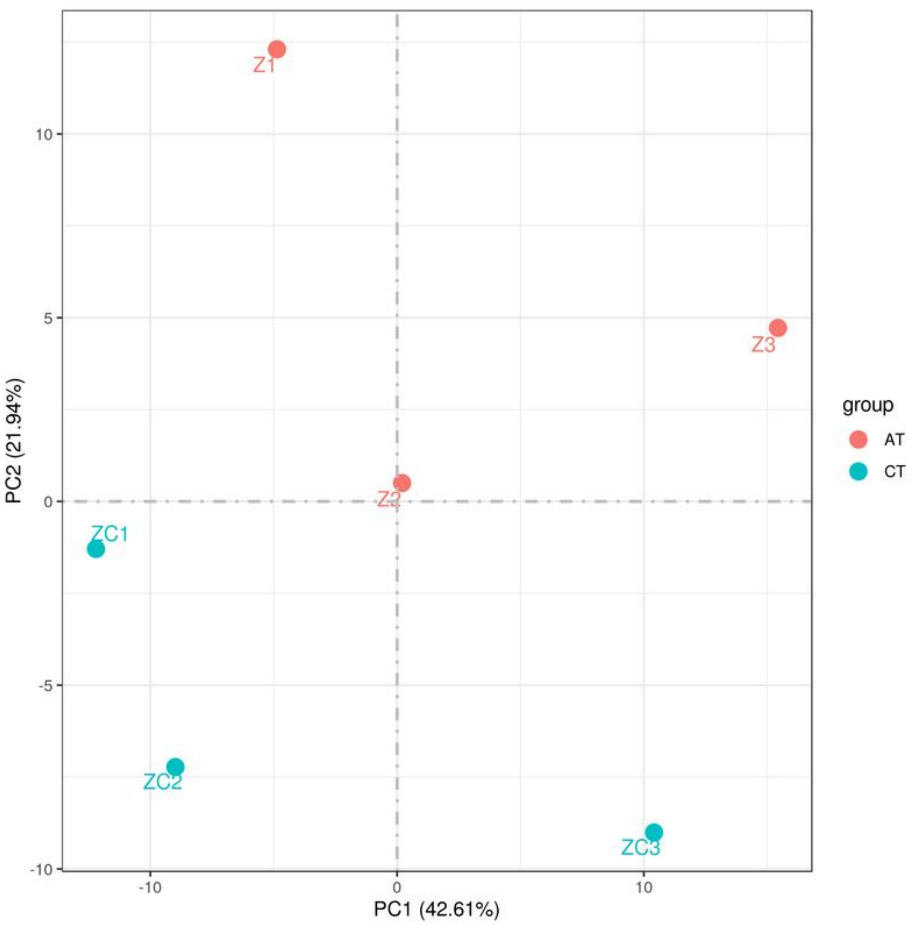

Figure S2. PCA analysis of Aspergillus flavus samples. Z1, Z2, and Z3 represented treatment samples, and ZC1, ZC2, and ZC3 represented control samples.
