# Supplementary figures and images for: Transcriptome and metabolome profiling reveal the inhibitory effects of food preservatives on pathogenic fungi
Source: PeerJ. 2025 Jul 23;13:e19737. doi: 10.7717/peerj.19737 (PMC12296564; doi:10.7717/peerj.19737)

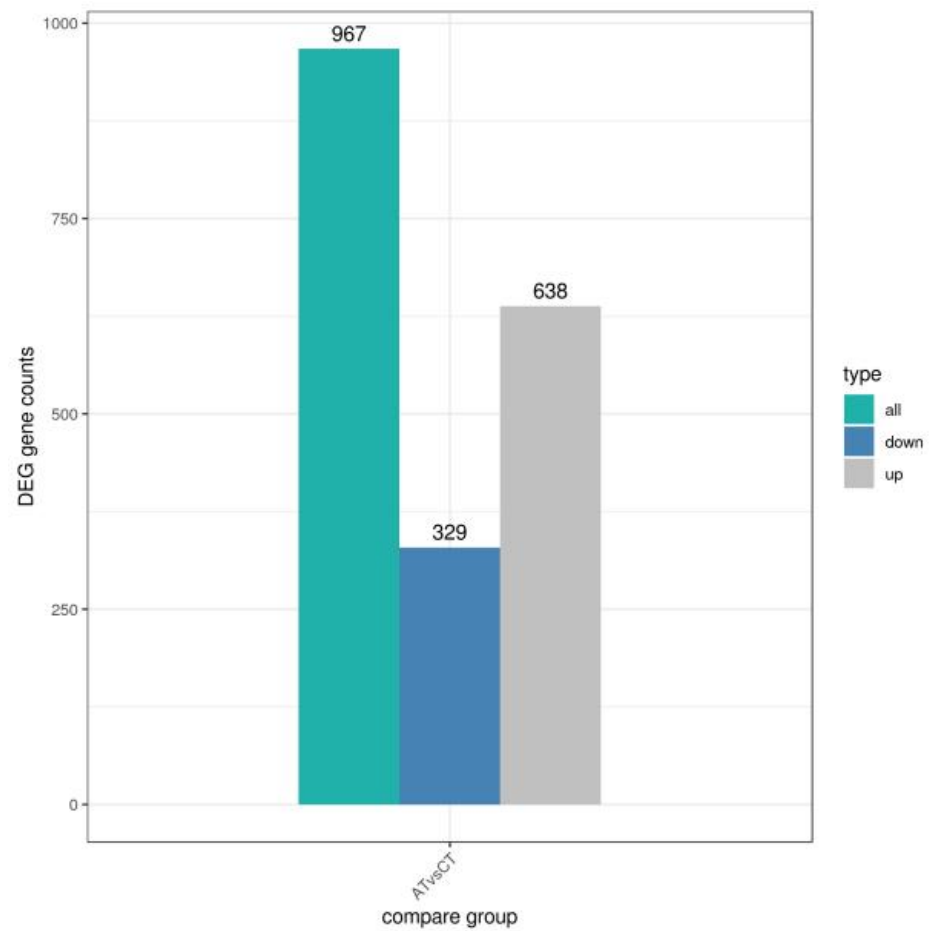

Figure S3. Analysis of differentially expressed genes in *Aspergillus flavus* samples.

Supplement: Supplemental Information 3 [file peerj-13-19737-s003.pdf]

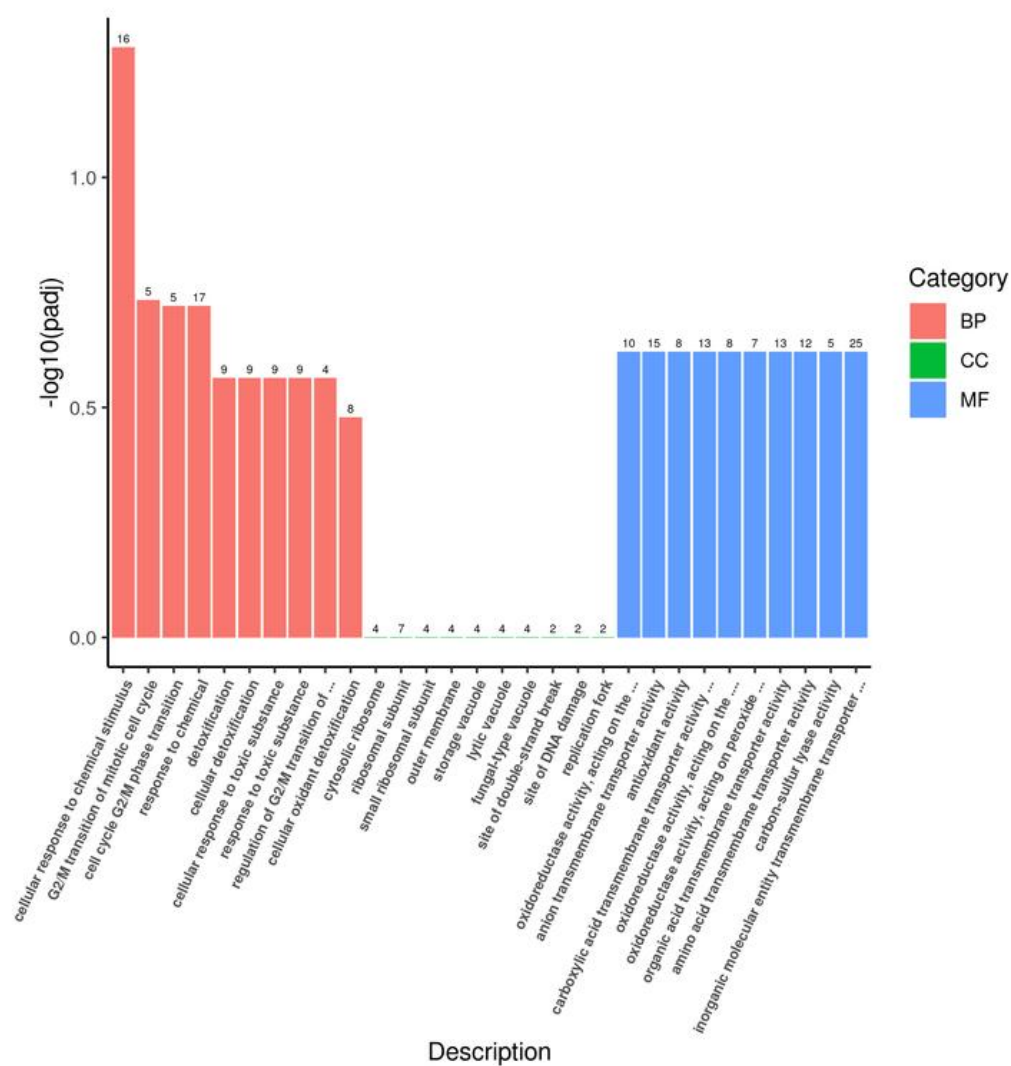

Figure S4. GO annotation classification of differentially expressed genes (DEGs).

Supplement: Supplemental Information 4 [file peerj-13-19737-s004.pdf]
