# Supplementary material for: Transcriptome and metabolome profiling reveal the inhibitory effects of food preservatives on pathogenic fungi
Source: PeerJ. 2025 Jul 23;13:e19737. doi: 10.7717/peerj.19737 (PMC12296564; doi:10.7717/peerj.19737)
Supplement: Supplemental Information 5 — The abscissa is the GeneRatio, the proportion of genes annotated in that entry to all DEGs, and the ordinate is each pathway entry. The size of the dots represents the number of annotated DEGs in the pathway, and the color of the dots represents the p-value of the hypergeometric test. [file peerj-13-19737-s005.pdf]

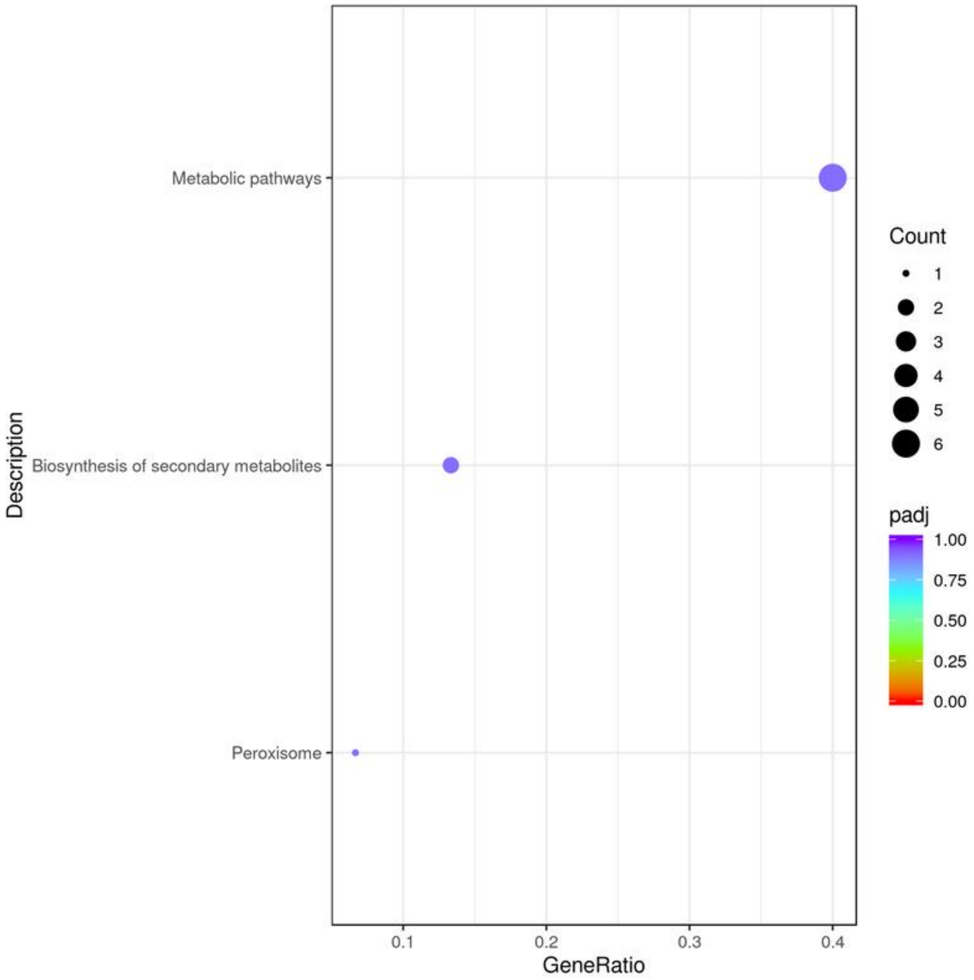

Figure S5. KEGG enrichment of differentially expressed genes (DEGs). The abscissa is the GeneRatio, the proportion of genes annotated in that entry to all DEGs, and the ordinate is each pathway entry. The size of the dots represents the number of annotated DEGs in the pathway, and the color of the dots represents the p-value of the hypergeometric test.
