# Supplementary material for: Transcriptome and metabolome profiling reveal the inhibitory effects of food preservatives on pathogenic fungi
Source: PeerJ. 2025 Jul 23;13:e19737. doi: 10.7717/peerj.19737 (PMC12296564; doi:10.7717/peerj.19737)
Supplement: Supplemental Information 6 [file peerj-13-19737-s006.docx]

**Table S1.** Primers for real-time quantitative PCR

| Target gene | Primer sequence (5’-3’) |
| --- | --- |
| AFLA_053390 | F: TCCTCATTCCCATGGCCAAC |
| AFLA_053390 | R: TCGCACATGTTGGTAGCTGT |
| AFLA_121370 | F: AAAGAGTGGGGCACTTCGAG |
| AFLA_121370 | R:GTTGGCCATGGGAATGAGGA |
| AFLA_024930 | F:GCGAATTGCAGCTGAGAAGG |
| AFLA_024930 | R: TAGAGCAGGTGCCGTTCTTG |
| AFLA_041970 | F:CCCTAGATCCGTAGCCTGGA |
| AFLA_041970 | R: GGATGAGCATATCACGGCCA |
| AFLA_002830 | F:TGCAGCTGAGAAGGACACTG |
| AFLA_002830 | R:GTGCCGTTCTTGTAGCGTTC |
| AFLA_030450 | F:CGACGACACGTAGTATCCCG |
| AFLA_030450 | R:CCTCCAGTGTTCACCAGCAA |
| AFLA_nor | F: GTCCAAGCAACAGGCCAAGT |
| AFLA_nor | R: TCGTGCATGTTGGTGATGGT |
