# Supplementary material for: Transcriptome and metabolome profiling reveal the inhibitory effects of food preservatives on pathogenic fungi
Source: PeerJ. 2025 Jul 23;13:e19737. doi: 10.7717/peerj.19737 (PMC12296564; doi:10.7717/peerj.19737)
Supplement: Supplemental Information 7 — Y represents the inhibition rate of fungal growth and X represents the concentration of preservative. [file peerj-13-19737-s007.docx]

Table S2. Inhibition equations of preservatives against *Aspergillus flavus*, *Alternaria alternata* and *Talaromyces funiculosus*. Y represents the inhibition rate of fungal growth and X represents the concentration of preservative.

| Preservatives | Strain | Toxicity regression equation | Correlation coefficient | Inhibition of medium concentration *EC*_50_ value (mg/ml) |
| --- | --- | --- | --- | --- |
| Sec-butamide | *A. flavus* | $y=3.4132x+3.9668$ | 0.7676 | 2.0077 |
|  | *A. alternata* | $y=4.2564x+4.4168$ | 0.8838 | 1.0709 |
|  | *T. funiculosus* | $y=8.7130x-1.3727$ | 0.8370 | 5.3876 |
| Citric acid | *A. flavus* | $y=8.9325x-2.1321$ | 0.8837 | 6.2869 |
|  | *A. alternata* | $y=8.9035x-1.0454$ | 0.9036 | 4.7753 |
|  | *T. funiculosus* | $y=7.8932x-0.8856$ | 0.8850 | 5.5674 |
| Potassium sorbate | *A. flavus* | $y=5.5466x+6.7583$ | 0.9900 | 0.4819 |
|  | *A. alternata* | $y=3.661x+4.7045$ | 0.9101 | 1.2042 |
|  | *T. funiculosus* | $y=3.9060x+3.8920$ | 0.8790 | 1.9216 |
