# Supplementary material for: Transcriptome and metabolome profiling reveal the inhibitory effects of food preservatives on pathogenic fungi
Source: PeerJ. 2025 Jul 23;13:e19737. doi: 10.7717/peerj.19737 (PMC12296564; doi:10.7717/peerj.19737)
Supplement: Supplemental Information 8 — Z1, Z2, and Z3 represented treatment samples, and ZC1, ZC2, and ZC3 represented control samples. [file peerj-13-19737-s008.docx]

**Table S3.** Quality control data for transcriptome sequencing. Z1, Z2, and Z3 represented treatment samples, and ZC1, ZC2, and ZC3 represented control samples.

| sample | library | raw_reads | raw_bases | clean_reads | clean_bases | Q30 | GC_pct |
| --- | --- | --- | --- | --- | --- | --- | --- |
| Z1 | FRAS230358043-1r | 47160536 | 7.07G | 46536774 | 6.98G | 95.28 | 53.05 |
| Z2 | FRAS230358044-1r | 42496024 | 6.37G | 42113970 | 6.32G | 94.86 | 51.58 |
| Z3 | FRAS230358045-1r | 47773864 | 7.17G | 47362386 | 7.1G | 94.87 | 52.38 |
| ZC1 | FRAS230358046-1r | 46033354 | 6.91G | 45603360 | 6.84G | 94.98 | 52.13 |
| ZC2 | FRAS230358047-1r | 46912616 | 7.04G | 45965310 | 6.89G | 94.64 | 51.18 |
| ZC3 | FRAS230358048-1r | 43299122 | 6.49G | 42612650 | 6.39G | 94.78 | 51.47 |
