# Supplementary material for: Transcriptome and metabolome profiling reveal the inhibitory effects of food preservatives on pathogenic fungi
Source: PeerJ. 2025 Jul 23;13:e19737. doi: 10.7717/peerj.19737 (PMC12296564; doi:10.7717/peerj.19737)
Supplement: Supplemental Information 9 [file peerj-13-19737-s009.docx]

**Table S4.** Statistical analysis of sequence alignment between sequencing data and reference genome.

| **sample** | **total_reads** | **total_map** | **unique_map** | **multi_map** |
| --- | --- | --- | --- | --- |
| Z1 | 46536774 | 43354982(93.16%) | 43132733(92.69%) | 222249(0.48%) |
| Z2 | 42113970 | 38741188(91.99%) | 38504929(91.43%) | 236259(0.56%) |
| Z3 | 47362386 | 44147357(93.21%) | 43947400(92.79%) | 199957(0.42%) |
| ZC1 | 45603360 | 42458854(93.1%) | 42204982(92.55%) | 253872(0.56%) |
| ZC2 | 45965310 | 42371153(92.18%) | 42085256(91.56%) | 285897(0.62%) |
| ZC3 | 42612650 | 39452908(92.58%) | 39268477(92.15%) | 184431(0.43%) |
